# Transcriptomic analysis identifies the key genes involved in stamen petaloid in lotus (*Nelumbo nucifera*)

**DOI:** 10.1186/s12864-018-4950-0

**Published:** 2018-07-27

**Authors:** Zhongyuan Lin, Rebecca Njeri Damaris, Tao Shi, Juanjuan Li, Pingfang Yang

**Affiliations:** 1 Key Laboratory of Plant Germplasm Enhancement and Specialty Agriculture, Wuhan Botanical Garden, Chinese Academy of Sciences, Wuhan, 430074 China; 2 University of Chinese Academy of Sciences, Beijing, 100039 China; 3 Sino-African Joint Research Center, Chinese Academy of Sciences, Wuhan, 430074 China

**Keywords:** Flower morphology, *Nelumbo nucifera*, Stamen Petaloid, Transcriptome analysis, Floral organ

## Abstract

**Background:**

Flower morphology, a phenomenon regulated by a complex network, is one of the vital ornamental features in *Nelumbo nucifera*. Stamen petaloid is very prevalent in lotus flowers. However, the mechanism underlying this phenomenon is still obscure.

**Results:**

Here, the comparative transcriptomic analysis was performed among petal, stamen petaloid and stamen through RNA-seq. Using pairwise comparison analysis, a large number of genes involved in hormonal signal transduction pathways and transcription factors, especially the MADS-box genes, were identified as candidate genes for stamen petaloid in lotus.

**Conclusions:**

Taken together, these results provide an insight into the molecular networks underlying lotus floral organ development and stamen petaloid.

**Electronic supplementary material:**

The online version of this article (10.1186/s12864-018-4950-0) contains supplementary material, which is available to authorized users.

## Background

Lotus (Nelumbo), a basal eudicot in the family of Nelumbonaceae, is a perennial aquatic plant, comprising of two species *Nelumbo nucifera* Gaertn. and *Nelumbo lutea* Pers.. Its relative simple genome size (about 929 Mb) makes it ideal for cross breeding and genetic studies [1, 2]. The *N. nucifera* is mainly cultivated in Asia, especially in China as an important horticultural crop with very high economic value, and it has been cultivated for more than 2000 years [3, 4]. The sacred lotus can be divided into three groups according to their agronomic traits and primary utilization, namely, rhizome, seed, and flower lotus.

Flower lotus are widely cultivated for their aesthetic value. The color and shape of its flower are the two major ornamental features, which largely determine its ornamental values. The aspect of flower shape includes the number, size, and shape of the petals. There are four distinct organ types (sepals, petals, stamens, and carpels) that commonly constitute a flower and are arranged in different whorls. For the purpose of ornamentation, petaloid is one of the characteristics selected in ornamental plants breeding [5]. Based on previous studies on the ranuncukid genus *Aquilegia*, a model system for studying the evolution of petals [6], there are two major types of petaloid. The andropetaloidy, which originates from a modified stamen, and the other is derived from pre-existing sterile bracts variation [7]. In lotus, the flower morphologies are classified as a few-petalled group, double-petalled group, duplicate-petalled group and all-double-petalled group [3]. Several lotus cultivars have novel petaloid differentiation, such as the stamen petaloid and carpel petaloid. Lotus flowers exhibit numerous transition petal shapes with some having a stamen appendage, indicating that the petals may belong to andropetals. There are distinct stamen petaloid cultivars including ‘Fenhonglingxiao’, ‘Zhigaowushang’ and ‘Manjianghong’. However, the genetic pathways controlling this phenomenon of stamen petaloid remain unclear in lotus.

Due to its importance for both horticultural ornament and plant reproduction, floral patterning has been well studied, particularly in *Arabidopsis* and *Antirrhinum*. Based on previous studies, genetic pathways controlling petal organogenesis in *Arabidopsis* have been elucidated and the molecular mechanism of petal identity relies on specialized signal transduction [8, 9]. Majority of the identified genes involved in these processes encode hormone pathway or transcription factors [8, 10].

Numerous studies focusing on floral organ identity genes have been conducted, providing the well-known ‘ABCE model’ of flower development [11–16]. In *Arabidopsis*, A class genes include *AP1* (*APETALA 1*) and *AP2* (*APETALA 2*); B class genes includes *AP3* (*APETALA3*) and *PI* (*PISITTALA*); C class contains only the *AGAMOUS* (*AG*); whereas, E class consist of four *SEPALLATA* (*SEP1*, *2*, *3*, *4*) members. The above genes are MADS-box genes except for *AP2* [11, 17]; with ‘quartet model’ suggesting that they interact with each other to form tetrameric complexes [15, 18]. Class A and E genes together with those in class B control petal development, with stamens being specifically controlled by class B genes in combination with those in class E and C [19]. Over the time, it has been widely believed for a long time that not all petals are homologous [20]. Loss of class C gene perhaps leads to the transition of the stamen to petal. Above-mentioned enlightenments indicate that the morphology of the stamen petaloid may be regulated by a complicated genetic pathway. However, it is still unknown whether this “ABCE” is also applicable in other plant species. Recently, several studies were performed using RNA-Seq to characterize the key genes controlling petal development in some non-model plant species, such as roses, *Brassica rapa* and *chrysanthemum* [21–23].

In lotus, the genome of an ancient lotus cultivar ‘China Antique’ has been sequenced and annotated [1], which facilitates studies of this plant on different aspects of molecular biology. Owing to the available genomic information, some transcriptomics studies have also been conducted on its rhizome growth and development, leaf development and flowering time [24–27]. However, there are still no studies conducted to explore its floral organ development. In the present study, to elucidate the mechanism of floral organ formation, especially the stamen petaloid, we conducted a comparative transcriptomic analysis among petal (P), stamen petaloid (Sp) and stamen (St) from an ornamental lotus cultivar ‘Fenhonglingxiao’ through RNA-seq technique. The results showed that several candidate genes might be involved in stamen petaloid in lotus.

## Results

### Stamen petaloid phenotype of *N. nucifera*

**Fig. 1:**
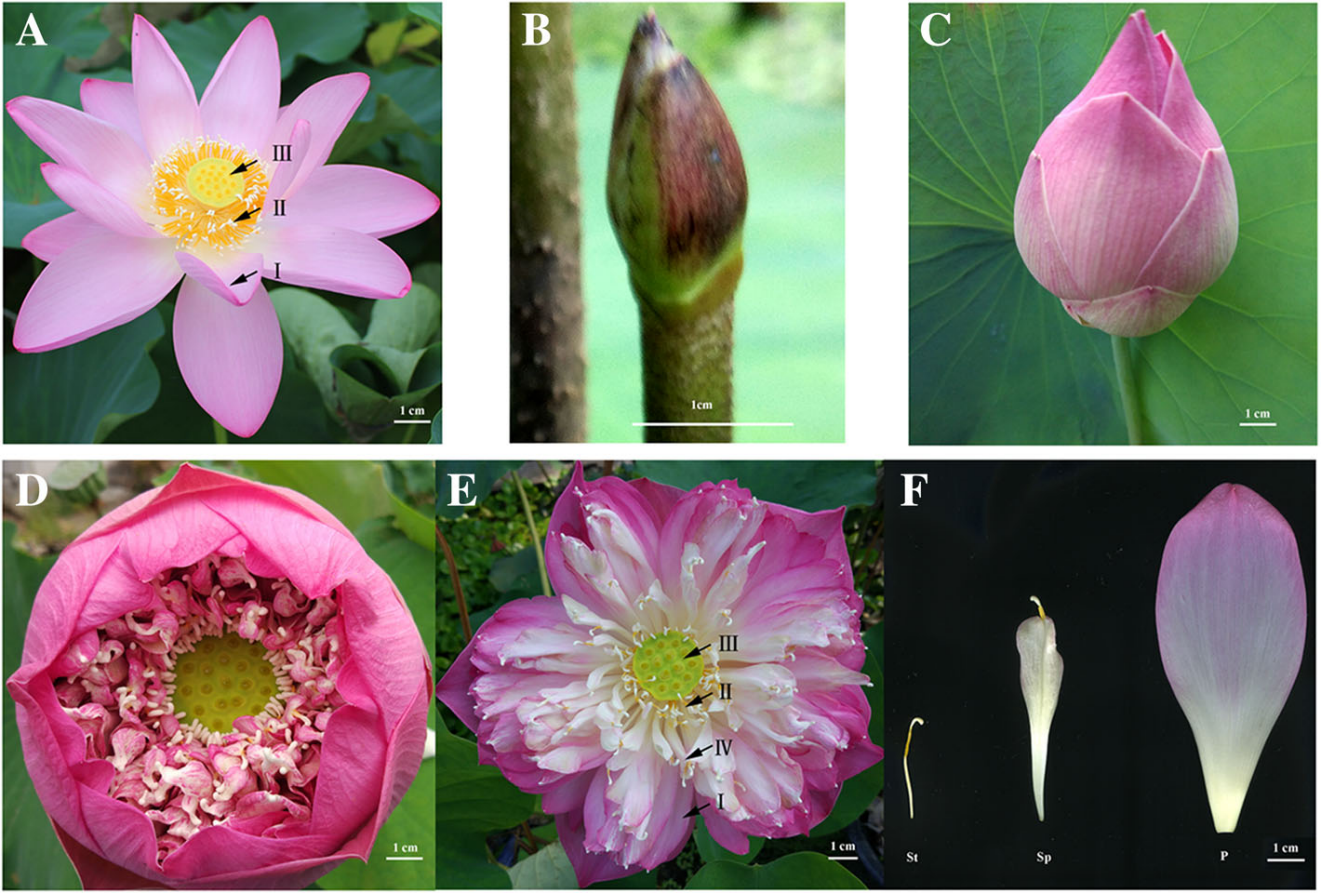
Sacred lotus flower. **a** Flower of ‘China antique’. **b** and (**c**) Flower bud of ‘Fenhonglingxiao’. **d** Flower of ‘Fenhonglingxiao’ at the first day of blossom. **e** Flower of ‘Fenhonglingxiao’ at the fourth day of blossom. Irepresents Petal (P); IIrepresents Stamen (St); III represents carpel; IV represents Stamen petaloid (Sp). **f** Three floral organs, including St, Sp, and P. Bars are all 1 cm

Flower shape is one of the important factors that determine the ornamental value of lotus. A normal flower contains four distinct organs, including sepals, petals, stamens, and carpels. The former three contribute greatly to the flower appearance and thus its ornamental value. As for lotus, the sequenced cultivar ‘China Antique’ contains normal petals, stamens, and carpels (Fig. 1a). For ornamentation, a series of flower lotus cultivars with different flower shape have been bred. Among them is the ‘Fenhonglingxiao’, a tropical lotus with floral aberration, in which stamens are converted to petal-like organs (Fig. 1b-f). Compared with ‘China Antique’, ‘Fenhonglingxiao’ has more petals and fewer stamens and the abnormal floral organ is known as stamen petaloid, which include stamen appendage being similar to petal (Fig. 1f). This feature provides a good model for the study of stamen petaloid.

### Illumina HiSeq mRNA sequencing

To further investigate the molecular mechanisms underlying the stamen petaloid phenotype, comparative transcriptomic analysis among the petal (P), stamen petaloid (Sp), stamen (St) through RNA-Seq was conducted. The floral organs, including P, Sp, and St, were sampled when the flower bloomed on the first day (Fig. 1d), and were used to construct nine independent libraries: P1, P2, P3, Sp1, Sp2, Sp3, St1, St2, St3, with digital number representing the three biological replicates.

**Fig. 2:**
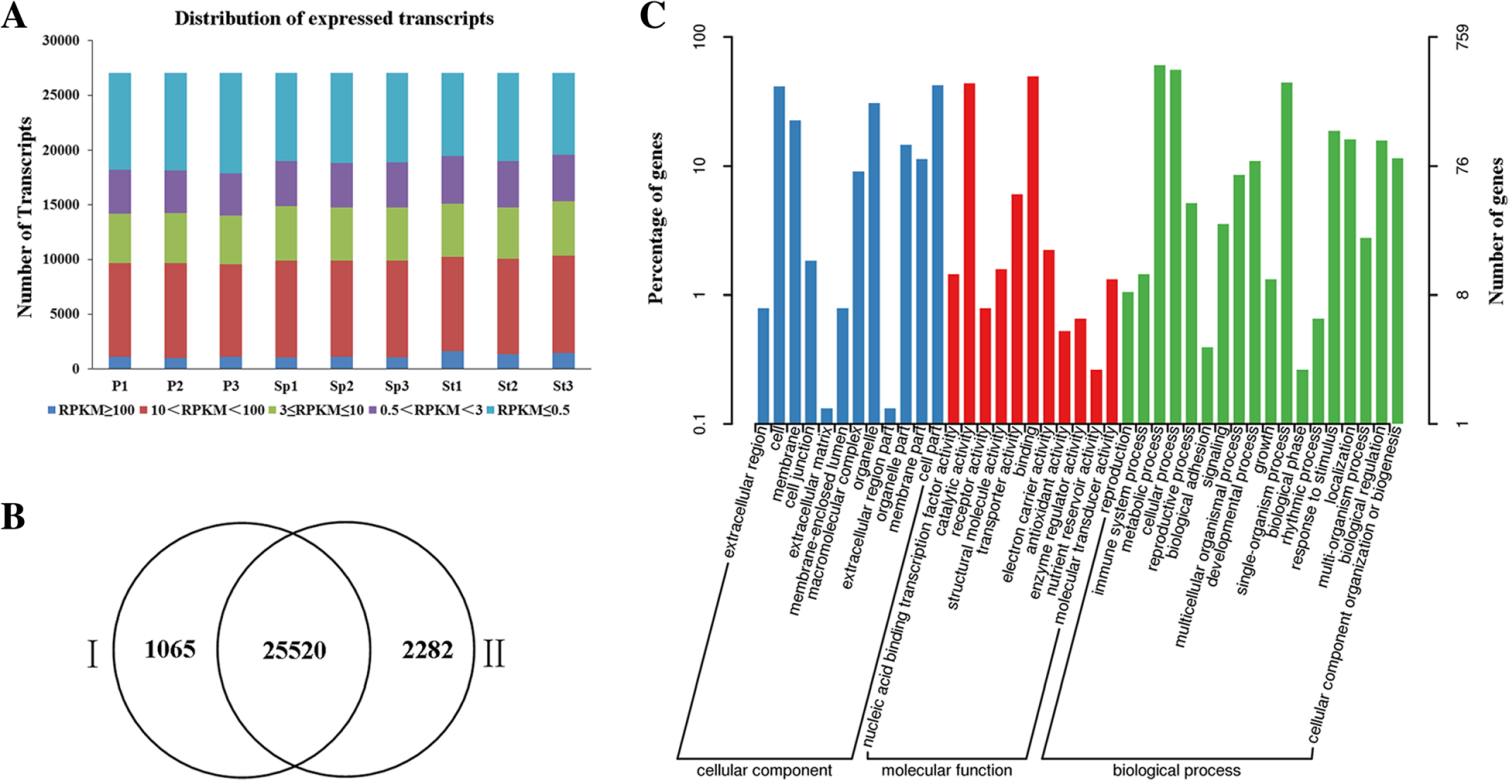
Information of the identified transcripts. **a** Abundance distribution of the expressed transcripts. **b** Venn diagram showing the overlap between the annotated genes from the lotus database (I) and the identified transcripts (II). **c** GO annotation of the novel genes

A total of 75.35 Gb clean data were generated from the nine libraries, with 73.11Gb high-quality (Q > 20) reads being selected, with each sample having over 6.51 Gb clean data used for further analysis. The number of transcripts identified in each sample, expressed in FPKMs (fragments per kilo base of transcript per million base pairs sequenced) are shown in Fig. 2a. Genes with normalized reads lower than 0.5 FPKM were removed from the analysis. In total, 27,053 transcripts were detected in the samples. Approximately 33% of the expressed genes were in the 0.5–10 FPKM range, while 36% were in > 10 FPKM (Fig. 2a). About 82.72–86.46% of the short clean reads were aligned against the ‘China Antique’ reference genome [1]. Among which, 73.63–76.29% of the reads were mapped to exon, 6.45–9.21% to intron, and 16.59–17.67% to intergenic (Table 1). For each of the three sampled floral organs, the Pearson correlation coefficient analysis showed they had good reproducibility among the three biological replicates (Additional file 1: Figure S1).

**Table 1:** Summary statistics of clean reads in the transcriptomes of lotus

samples	Total Reads	Base number (G)	Q20 (%)	Mapped Reads	Exon (%)	Intron (%)	Intergenic (%)	GC (%)
P1	48,494,178	7.27	97.30	41,774,628 (86.14%)	73.63	9.21	17.16	44.93
P2	62,315,936	9.35	96.71	53,447,367 (85.77%)	74.02	9.04	16.94	44.86
P3	58,545,264	8.78	97.28	50,620,458 (86.46%)	73.79	8.8	17.41	44.68
Sp1	64,909,918	9.74	96.75	54,890,928 (84.56%)	75.62	7.79	16.59	45.26
Sp2	63,780,056	9.57	97.01	52,759,965 (82.72%)	74.94	8.32	16.74	45.22
Sp3	47,264,990	7.09	97.33	39,552,586 (83.68%)	75.02	8.2	16.79	45.13
St1	62,630,786	9.39	96.65	52,672,417 (84.10%)	75.31	7.02	17.67	45.62
St2	43,369,878	6.51	97.11	37,127,935 (85.61%)	75.17	7.33	17.5	45.35
St3	51,028,440	7.65	97.34	42,459,724 (83.21%)	76.29	6.45	17.26	46.17

Six databases (COG, GO, KEGG, KOG, SwissProt, Nr) were used to annotate all genes to provide comprehensive gene function information. Based on the annotation results (Table 2), 10,193 genes (36.66%) were annotated in COG; 21,434 genes (77.10%) in GO; 10,268 genes (36.93%) in KEGG; 14,775 genes (53.14%) in KOG; 20,768 genes (74.70%) in SwissProt and 27,784 genes (99.94%) in Nr. Altogether,27,802 genes were annotated. Moreover, 25,520 out of 27,802 genes were previously predicted in the lotus reference [1]. The rest 2282 genes could not be identified in the reference genes, representing novel genes (Fig. 2b). Of the 2282 novel transcripts, a total of 1836 genes were successfully annotated using all annotation databases (Additional file 1: Table S1). Also, 758 genes were annotated by GO assignments, being categorized into three major groups (cellular component, molecular function, and biological process), and 41 sub-categories (Fig. 2c**)**.

**Table 2:** All genes annotation

Annotated Database	Annotated Number	Percentage (%)	300 < =length < 1000	length > =1000
COG_Annotation	10,193	36.66	3901	5989
GO_Annotation	21,434	77.10	9137	11,082
KEGG_Annotation	10,268	36.93	4556	5037
KOG_Annotation	14,775	53.14	6292	7577
Swissprot_Annotation	20,768	74.70	8634	11,011
Nr_Annotation	27,784	99.94	12,150	13,716
All_Annotated	27,802	100.00	12,156	13,717

### Differentially expressed genes (DEGs) among floral organs

**Fig. 3:**
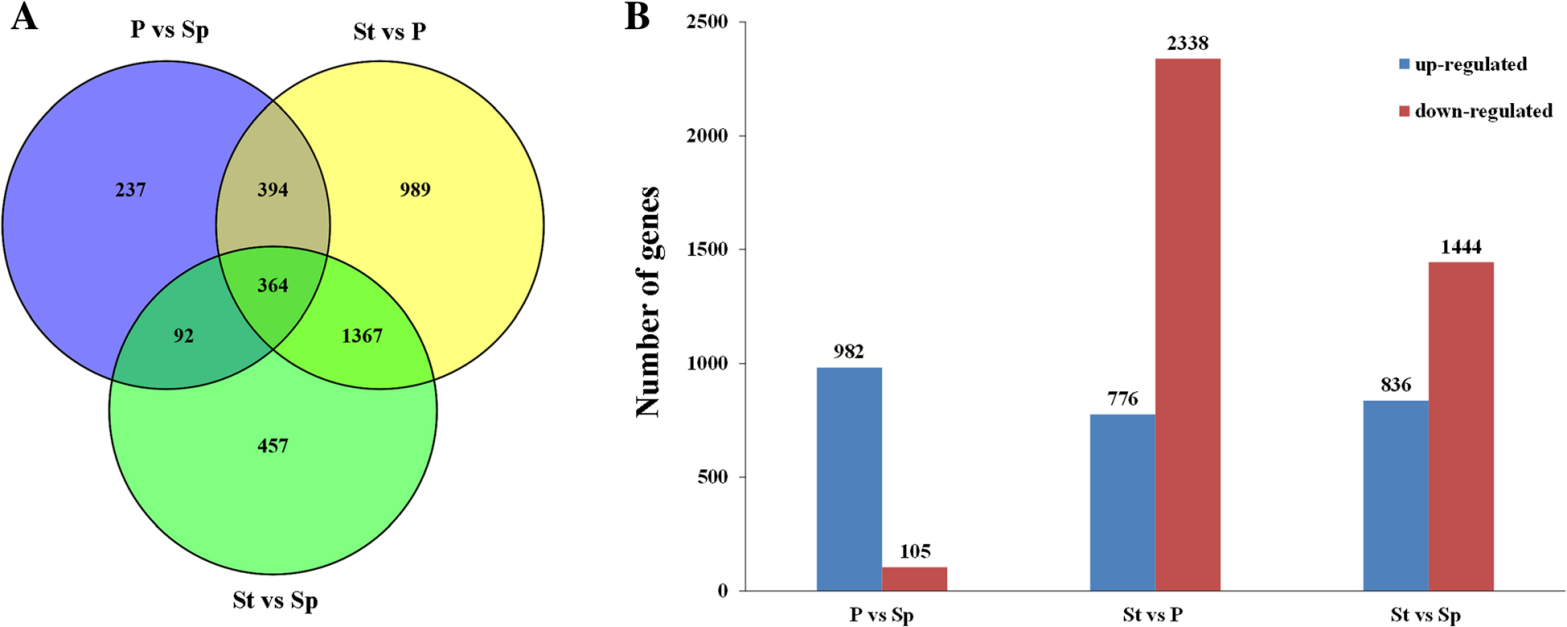
DEGs among the three floral organs. **a** Venn diagram of the number of unique and common DEGs among the comparisons between P vs Sp, St vs P and St vs Sp. **b** The number of up-regulated and down-regulated DEGs in the comparisons between P vs Sp, St vs P and St vs Sp. The y-axis is the number of genes, and the x-axis is the different comparison

To identify the genes associated with the stamen petaloid, we conducted pairwise comparison among petal, stamen petaloid and stamen (Fig. 3). The FPKM was used to estimate the level of gene expression and the expression levels were compared in each floral organ to identify significant DEGs, with a fold change of no less than two and a False Discovery Rate (FDR) < 0.05. In total, 3900 genes were differentially expressed among these three organs (Additional file 2). We found that there were 1087, 2280 and 3114 DEGs between P vs Sp, St vs Sp, and St vs P, respectively (Fig. 3a). Of these DEGs, 364 genes were significantly differentially expressed among the three floral organs. The overlapped number of DEGs detected from P vs Sp and St vs Sp was 456, which might be specifically involved in the stamen petaloid formation. Additionally, comparing with Sp, 631 and 1824 genes were specifically regulated in petal and stamen, respectively. Moreover, comparing of P vs Sp, 982 and 105 transcripts were up- and down-regulated, respectively (Fig. 3b), while in St vs P and St vs Sp, their up-regulated genes were both less than the down-regulated genes. In the St vs P comparison, there were 776 and 2338 transcripts up- and down-regulated, respectively, and in St vs Sp, the up-regulated genes were 836 with the down-regulated genes being 1444.

**Fig. 4:**
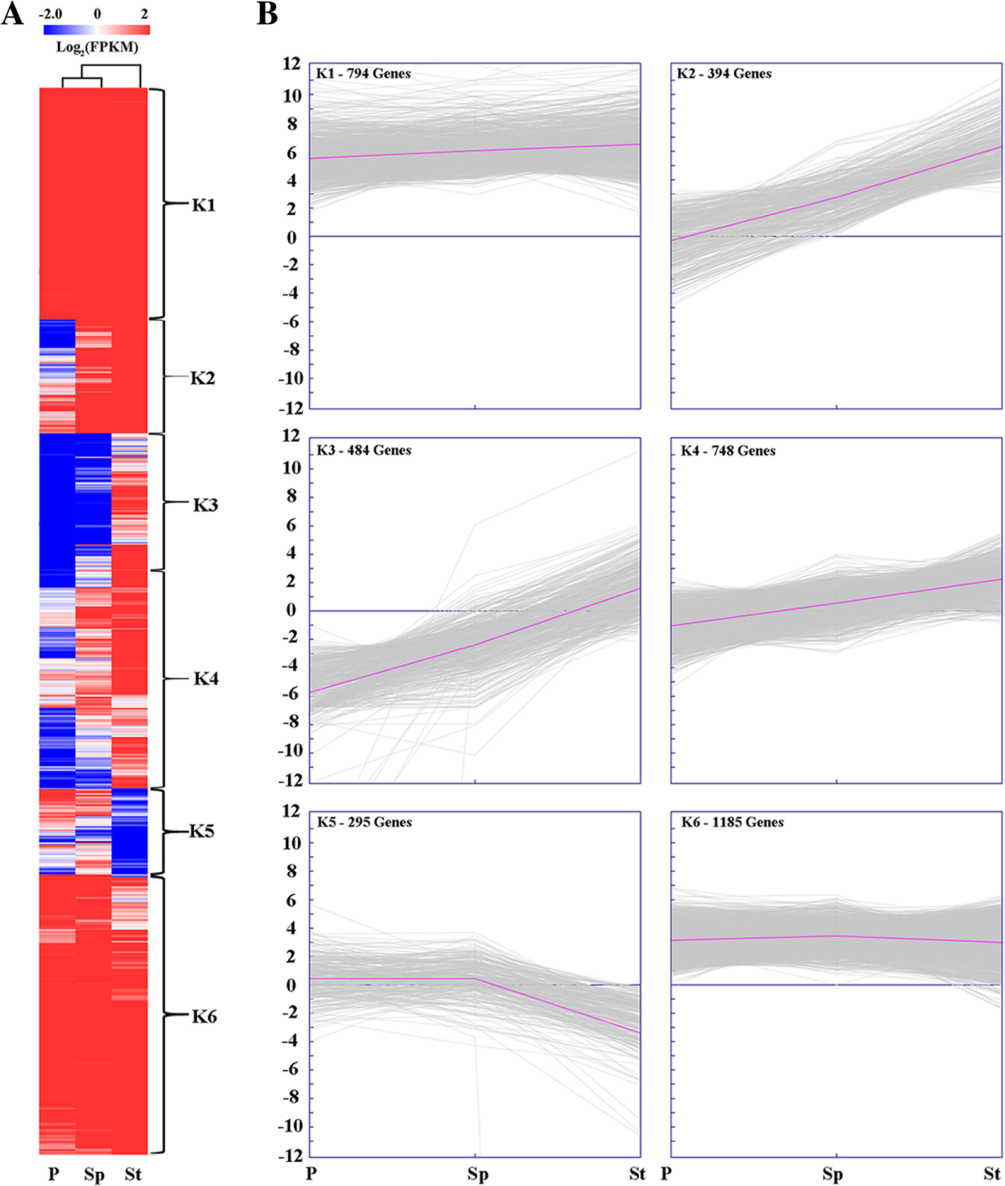
Expressional patterns of the DEGs identified among the three floral organs. **a** The heatmap of DEGs in three floral organs. FPKM (fragments per kilo base of transcript per million base pairs sequenced) normalized log_2_ transformed counts were used to estimate the level of gene expression. The red (high expression) and green (low expression) represents the relative expression level. **b** The nine main clusters with different gene expression pattern types, namely K1-K6. The numbers of DEGs in each cluster are displayed

The clustered patterns of all DEGs expression during floral development were created using the Euclidean distance method associated with complete-linkage (Fig. 4a and b). K-means cluster analysis plotted the DEGs into six distinct expression patterns, K1-K6 (Fig. 4b). The K1 and K6 cluster contained 794 and 1185 genes respectively that had no obvious change in three different floral organs. The genes in cluster K2, K3 and K4 showed a similar expression pattern. K2 and K3 clusters having 394 and 484 genes, respectively, had an above the zero transcription level, and genes in cluster k2 were significantly changed among the three floral organs. Meanwhile, 748 genes in K4 cluster had almost all genes expression levels being below zero, except some in the stamen. The K5 cluster including 295 genes had a specific pattern being the only cluster with decreased expression in stamen.

We used GO assignments to classify the functions of DEGs in pairwise comparisons of cDNA libraries between different floral organs (Additional file 1: Figure S2A). The GO enrichment revealed 20 categories of biological processes, 16 categories of cellular component and 17 categories of molecular function which had similar pattern in the DEGs and all genes among P vs Sp, St vs Sp and St vs P, except ‘structural molecular activity’ category in P vs Sp, whose percentage was not corresponding to the pattern (Additional file 1: Figure S2A). To further explore the functional classification and pathway assignment of DEGs, we performed KEGG analysis. Dozens of genes were enriched in phenylpropanoid biosynthesis, phenylalanine metabolism, flavonoid biosynthesis, pentose and glucoronateinter conversion, nitrogen metabolism, amino sugar and nucleotide sugar metabolism, ascorbate and aldarate metabolism, starch and sucrose metabolism, alpha-linolenic acid metabolism and photosynthesis (Additional file 1: Figure S2B).

### DEGs involved in stamen petaloid

**Fig. 5:**
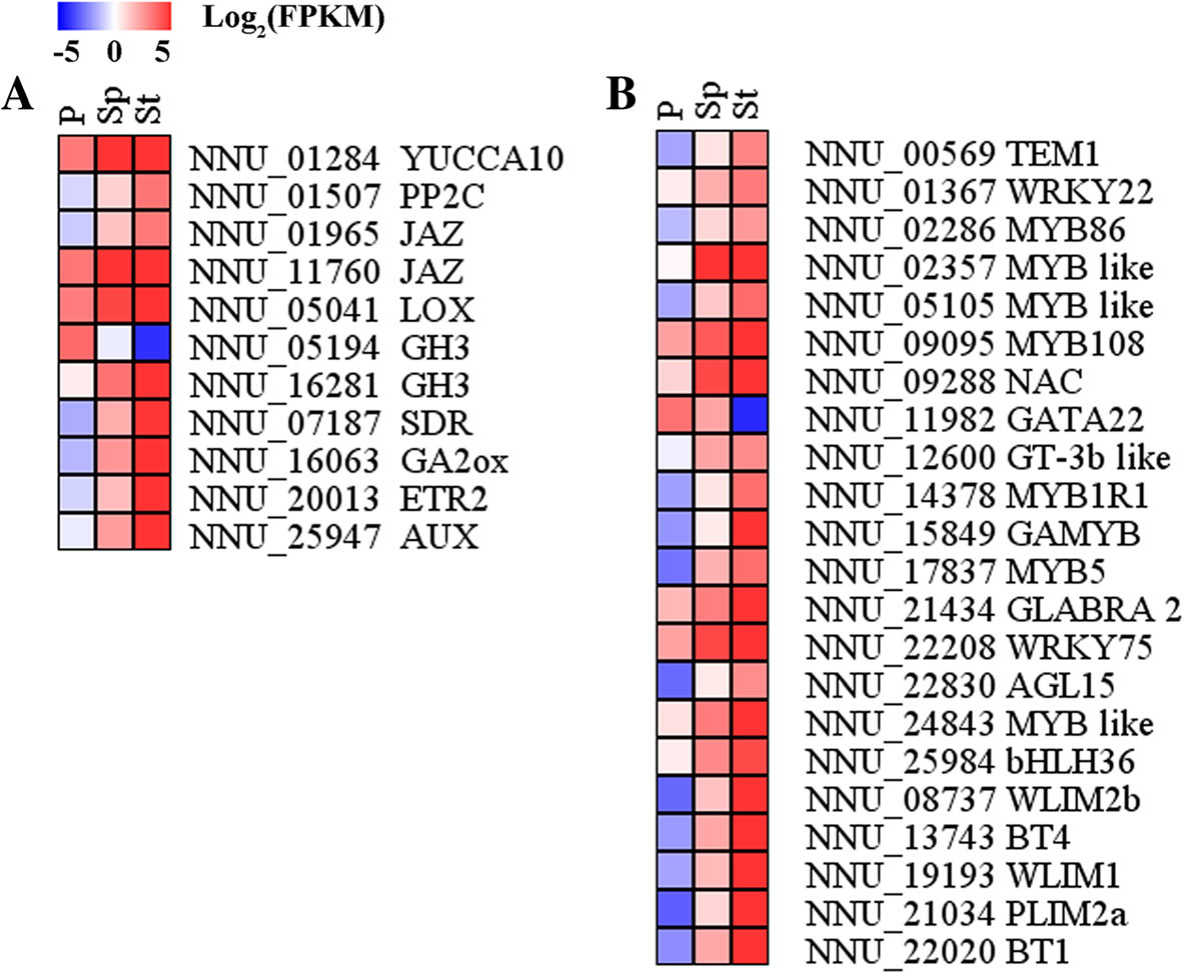
Heat maps of phytohormone-related genes and transcription factors (TFs) in comparison of three floral organs. **a** Eleven phytohormone-related genes. **b** 22 transcription factors

To elucidate the genetic regulation of the stamen petaloid formation phenomenon, the differentially expressed genes were filtered for those believed to be involved in stamen petaloid. As stated above, 364 DEGs, could be the specific genes through which stamen petaloid may be regulated. There were numerous genes assigned to phytohormone pathways, including the auxin (AUX), abscisic acid (ABA), jasmonic acid (JA), cytokinine (CTK), gibberellin (GA), brassinosteroid (BR), ethylene (ETH), and salicylic acid (SA) pathways. The transcriptome annotated as being involved in known phytohormone biosynthesis and signaling pathways are displayed in Additional file 3 with DEGs in the different floral organ presented in Additional file 1: Figure S3. Eleven out of 364 DEGs were annotated as being related to phytohormone (Fig. 5a and Additional file 1: Figure S4). Four of them were involved in AUX, namely, *YUCCA* (NNU_01284), *AUX* (NNU_25947), and *GH3* (NNU_16281 and NNU_05194). The protein phosphatase 2C (PP2C, NNU_01507) and one short-chain dehydrogenase/reductase (SDR, NNU_07187) were shown to be involved in the ABA pathway. Three genes encoding the jasmonate ZIM domain-containing protein (JAZ, NNU_01965 and NNU_11760), the lipoxygenase (LOX, NNU_05041) involved in the JA pathway, GA biosynthesis gene encoding for gibberellin 2-beta-dioxygenases (GA2ox; NNU_16063), and protein EIN4-like (ETR, NNU_20013) involved in ETH pathway, considerably varied in expression in the three floral organs and peaked in stamen. The expression patterns of ten out of eleven genes in three organs were similar, while NNU_05194 had an opposite pattern (Fig. 5a and Additional file 1: Figure S4). However, all of them exhibited a medium expression level in stamen petaloid. The expression of BR, SA, and CTK pathway genes (not listed above) also showed varied expression among the three samples.

A large number of DEGs were transcription factors (TFs) encoding genes. In our data, there were 89 TFs in P vs Sp, 172 TFs in St vs P, and 102 TFs in St vs Sp with the total number of TFs identified being 225 (Additional file 1: Figure S5). In the pair comparison of the three floral organs, there were 22 TFs amongst the 364 DEGs. 21 TFs, including eight MYBs like (NNU_02286, NNU_02357, NNU_05105, NNU_09095, NNU_14378, NNU_15849, NNU_17837 and NNU_24843), three putative homologs of LIM family members (NNU_08737, NNU_19193, and NNU_21034), two transcripts encoding WRKY (NNU_01367 and NNU_22208), two homologs of BTB/POZ and TAZ domain-containing proteins (BT: NNU_13743 and NNU_22020), one transcript encoding bHLH (NNU_25984), AP2/ERF and B3 domain–containing TEM1 (NNU_00569), Homeobox-leucine zipper protein GLABRA 2 (NNU_21,434), trihelix transcription factor GT-3b-like (NNU_12600), one homolog of NAC (NNU_09288), and AGAMOUS-like MADS-box protein AGL15 (NNU_22830) showed a down-regulation change in St compared with P or Sp. Nevertheless, only one TFs (GATA22, NNU_11982) was up-regulated in St vs P (Fig. 5b and Additional file 1: Figure S6). Similar to the hormone pathway genes, they also had a medium expression level in stamen petaloid. In addition, the homolog of NAC (NNU_09288) and AGL15 (NNU_22830) were down-regulated with more than a five-fold threshold. The MADS-box family members are key regulators of floral organ identity [28]. We regard the functions of MADS-box with extremely high interest.

In total, there were 12 members of floral homeotic protein, including 11 MADS-box TFs and one *APETALA2* (NNU_17043) observed in our data (Additional file 1: Figure S7 and Additional file 4). The MADS-box family members have been reported to be involved in the floral development [15]. Seven floral homeotic protein encoding genes exhibited the substantial changes in each pairwise comparison with a medium expression level in stamen petaloid (Additional file 1: Figure S7 and Additional file 4). Similar to NNU_22830, the *AGL15* homolog gene (NNU_01653), two homologs of floral homeotic protein AGAMOUS (NNU_10192 and NNU_26656), and the homolog of *AGL80* (NNU_09213) were down-regulated in St vs P. Nonetheless, the expression levels of two AGL6 like (NNU_08847 and NNU_26581) were up-regulated in the same pairwise comparison.

### Confirmation of gene expression through qRT-PCR

To assess the quality of the RNA-seq data, a total of 29 genes, including 12 homeotic floral genes (11 MADS-box genes and one *AP2*), were selected as representatives of specific DEGs for qRT-PCR (Additional file 1: Figure S8). The specific primers are listed in Additional file 1: Table S2. We showed that 29 genes had significant correlations between qRT-PCR result and RNA-Seq data and thus providing guaranteed data reliability (Additional file 1: Figure S9).

**Fig. 6:**
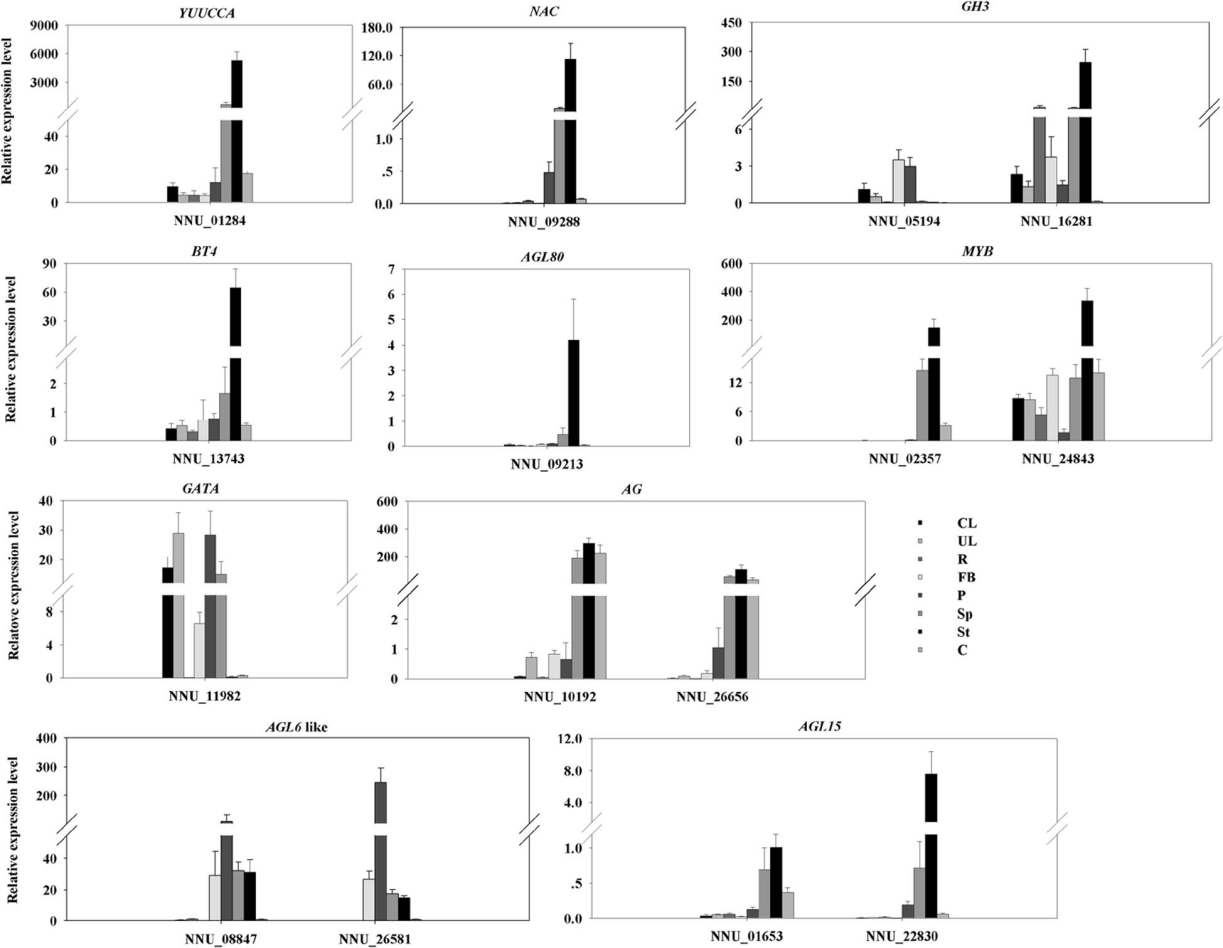
The expression pattern of fifteen candidate genes in lotus. CL: Curly Leaf; UL: Unfolded Leaf; Rhizome: R; FB: Flower Bud; Petal: P; Stamen petaloid: Sp; Stamen: St. Relative gene expressions were normalized by comparison with the expression of lotus β-actin (NNU_24,864), and using the 2^-ΔΔCT^ method. Each gene used three biological replicates, with three technical replicates per experiment in all qRT-PCRs. The Error bars represented the SD for three biological replicates

To validate the involvement of these gene in petaloid formation, three DEGs encoding AUX pathway, 12 transcription factors included seven MADS-box genes, were chosen to examine their gene expression profiles in eight tissues (curly leaf, unfolded leaf, rhizome, flower bud, petal, stamen petaloid, stamen, and carpel) (Fig. 6). Systematic analysis of the expression patterns of 15 candidate genes may reveal their morphologic roles in different tissues. Six genes, including *BT4* (NNU_13743), *YUUCCA10* (NNU_01284), *MYB* (NNU_02357), *NAC* (NNU_09288), *AGL80* (NNU_09213), and *AGL15* (NNU_22830), showed similar uniform expression patterns. Their expression levels showed higher enrichment in floral organs and peaked in stamen but were absent in vegetative tissues. *GH3* (NNU_16281) also showed similar expression profiles as these six genes, except for the transcript from rhizome. This indicates that NNU_16281 could also be essential for rhizome formation. However, *GH3* (NNU_05194) had a higher expression level in flower bud and petal and weakly expressed in other organs. *MYB* (NNU_24843) was mostly detected in stamen and lowly expressed in petal. In our result, *GATA* (NNU_11982) was the exclusive gene, which was expressed at high level in vegetative tissues (curly leaf and unfolded leaf but rhizome). Meanwhile, it also had enrichment in flower bud, petal, and stamen petaloid, and a lower level in stamen and carpel. Similarly, two *AGL6* like (NNU_08847 and NNU_26581) had high abundant in petal, with their transcript levels being higher in flower bud. Two *AG* (NNU_10192 and NNU_26656) had relatively higher expression levels in flower organs except for petal, while low expressions were detected in other organs. In this study, the spatial expression of all seven MADS-box genes significantly higher in floral organs compared to the low expression in vegetative organs (Fig. 6).

## Discussion

### Morphological and transcriptomic analyses showing the petal features of petaloid stamen

Floral morphology, formed by specific organs, specifically the petal, contributes to the ornamental values in flowering plants. Stamen petaloid has been frequently selected as one of the paramount ornamental features in flower lotus breeding. Here, we selected a lotus cultivar ‘Fenhonglingxiao’ showing stamen petaloid (Fig. 1). Based on the petal, stamen petaloid, and stamen morphology, it could be easily concluded that petaloid stamen lies in the middle of transition from stamen to petal. To investigate the mechanism underlying stamen petaloid in this cultivar, a comprehensive transcriptome analysis was conducted among the petal, stamen petaloid, and stamen. The number of DEGs detected in P vs Sp was much less than those in St vs P and St vs Sp (Fig. 3), suggesting that the petaloid stamen is more similar to petal than stamen, which was further supported by the Pearson correlation coefficient analysis (Additional file 1: Figure S1). The total number of identified genes was 27,802, a high value than the annotated genes in the lotus database. Although 1065 annotated genes were not identified, there were 2282 novel genes. Together with our previous study [26], it’s clear that more transcriptomic studies are necessary to optimize the lotus genome annotation.

### Involvement of phytohormones and transcription factors in stamen petaloid in lotus

Petals and stamens, being among the most important floral organs are located in the second and third whorls, respectively. The stamen petaloid grows between petal and stamen, and its morphology contains both of their features (Fig. 1a). This phenomenon may be regulated at the gene level as the petal spill out into the whorl of stamen or else the failure in expression of stamen identified genes. From the commonly expressed genes in St vs P, St vs Sp, and P vs Sp, genes controlling specific regulation in Sp were obtained.

Phytohormones are very crucial factors that affect organ genesis and development. Numerous genes involved in different phytohormone metabolism or signaling were differentially expressed among the three tissues. In this study, we specifically focused on the auxin pathway that controls floral organ formation. Auxin has been reported to play a crucial role in determining the identity of floral organs [29, 30]. In this work, auxin biosynthesis related gene *YUCCA10* (NNU_01284) had a high expression in floral tissues (Fig. 5 and Additional file 3), especially in stamen, indicating its critical role in the formation and development of floral organs in lotus. Previous studies reported that anthers are the major sites that produce high concentrations of auxin in *Arabidopsis* [31]. Auxin might be synthesized more in stamen than in petal, but then possibly transported into petal for its action. Several auxin signaling related genes, showed higher expression levels in petal and petaloid stamen than stamen (Additional file 1: Figure S3 and Additional file 3), which is consistent with previous study [23], indicating that auxin may promote the formation of petaloid stamen. However, one auxin signaling related gene *GH3* (NNU_16281) exhibited a predominant expression in stamen of lotus (Fig. 5 and Additional file 3). This result indicated that auxin signaling related genes have complicated function in regulation of floral organs formation.

**Fig. 7:**
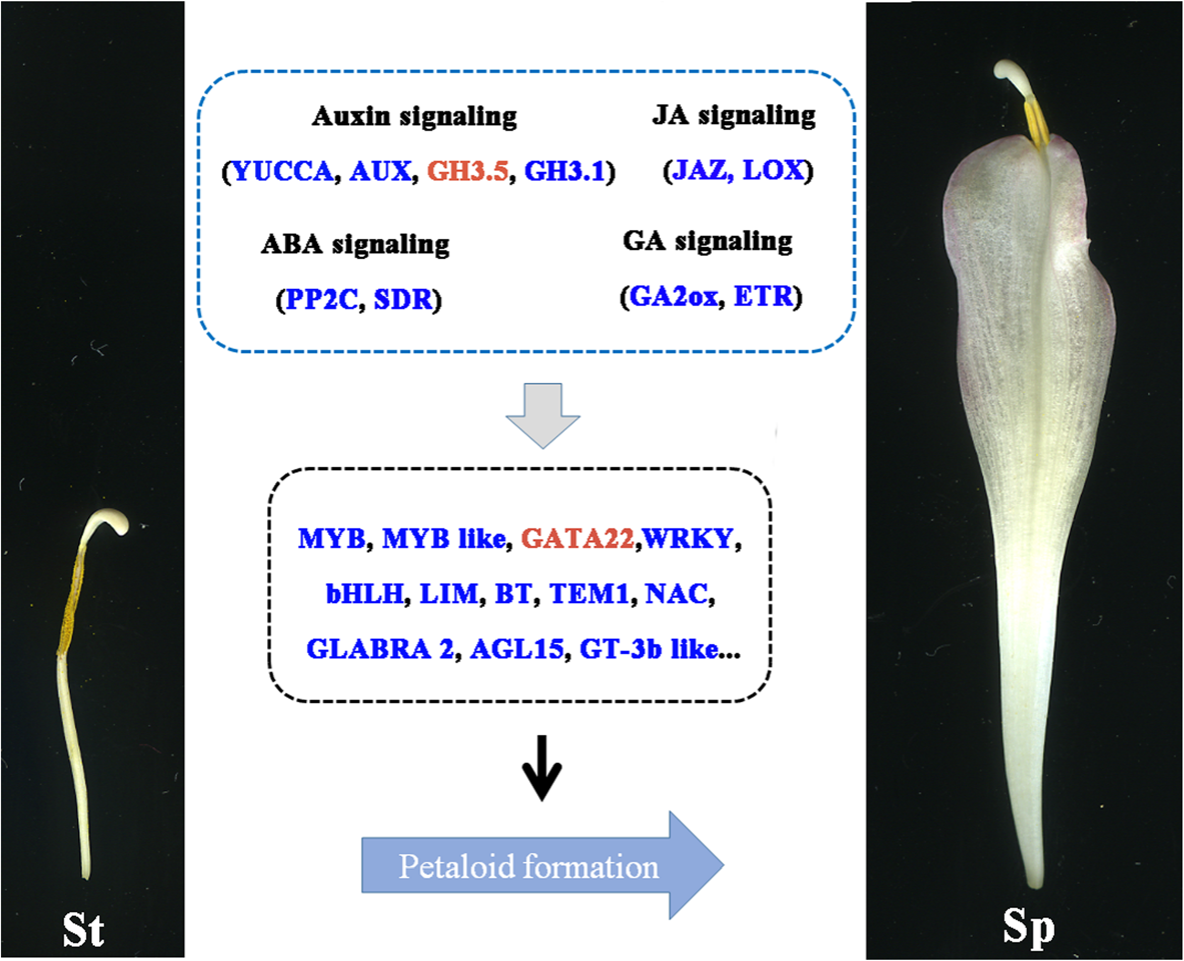
Summary of transcription-level regulation of stamen petaloid. Differentially expressed genes (DEGs) that may regulate stamen petaloid (those with more than a one-fold change in expression, FDR < 0.05). DEGs were either up-regulated (orange) or down-regulated (blue) as petaloid formation

The expression of numerous TFs changed drastically in lotus floral organs (Fig. 5 and Additional file 5). 225 TFs were identified as part of the DEGs and for further analysis, we focused on 22 TFs from the 364 DEGs obtained from the three organ comparison. Previously, it was reported that MYB is essential for floral organ development and plays a key role in pollen development in various plant species, such as rice, cotton and *Arabidopsis* [32–34]. In lotus, we have reported that there are potential 116 *MYB* genes in the lotus genome, with a number of them being related to flavonoid biosynthesis [35]. However, the mechanisms underlying floral morphologies are still obscure. Here, two genes encoding MYB (NNU_02357 and NNU_24843), showed a higher expression level in stamen and necessitates further investigation. It has been demonstrated that NAC transcription factor participates in floral-boundary morphogenesis in tomato [36]. NNU-09288, which was annotated as NAC-like TFs, probably participated in stamen development. However, NAC was also reported to control ethylene-regulated cell expansion in petals [37]. It has been shown that GATA transcription factors are negatively regulated by the floral homeotic genes *APETALA3* and *PISILLATA* in petals and stamens [38]. Similarly, a GATA3 family member *GNC* participated in stamen and carpel development [39]. HAN encoding a GATA transcription factor transcribes at the boundaries between different floral whorls, whose mutant diminished boundary formation, suggesting that it is involved in controlling cell differentiation [40]. The key role of HAN in determining petal number also was demonstrated [41]. In our study, a GATA-like gene (NNU_11982) had a higher expression in the petal compared to stamens and stamen petaloid, indicating its potential roles in breaking down the boundary of stamen and petal and hence promoting stamen conversion to petal-like. The DEGs probably involved in the conversion of stamen to stamen petaloid is summarized in Fig. 7.

Interestingly, we found fewer up-regulated DEGs than down-regulated DEGs in St vs Sp. After our critical selection, only two up-regulated DEGs were considered as the most potential candidates that cause the stamen petaloid formation, including auxin signaling related gene *GH3* (NNU_05194) and *GATA* (NNU_11982). A recent study reports that GH3.5 may be a direct target of *HAN* (a member of GATA family) and the functions of *HAN* as a key repressor that regulates floral development, along with genes involved in hormone action [39]. Some GATA family transcript factors integrate auxin, GA, and CTK signaling pathway to control plant development [41, 42]. GATA transcription factors play roles in multiple developmental courses. Thus, the possible interaction between the *GH3* (NNU_05194) and *GATA* (NNU_11982) could promote the change of stamen to stamen petaloid.

### Regulation of stamen petaloid by floral homeotic genes

Members of the MADS-box family are known to be involved in floral organ specification [43]. Transcription factors including 40 MADS-box genes in sacred lotus were identified [2]. In this study, we detected 12 floral homeotic genes, including 11 MADS-Box genes and one *AP2* (Additional file 4). Among them, seven MADS-box genes further analyzed through RT-qPCR on their expression pattern. We found that all of them had abundance expression in floral organs (Fig. 6). Previous studies have demonstrated that *AG* plays several key roles in floral organ determination, such as stamen identity and carpel specify [44–46]. In our study, two homologs of *AG*, NNU_10192 and NNU_26656 were both highly expressed in stamen petaloid and stamen (Fig. 6 and Additional file 1: Figure S8). The MADS-domain factors AGL15 like, NNU_22830 and NNU_01653 were most abundant in stamen; especially, the accumulation of NNU_22830 was down-regulated over five-fold in St vs P (Fig. 6 and Additional file 1: Figure S8). NNU_22830 was the only MADS-box gene common in St vs P, St vs Sp, and P vs Sp (Fig. 5). That indicated that NNU_22830 may play a vital function in stamen petaloid. Previous studies demonstrated that *AGL15* and *AGL18*, along with *SVP* and *AGL24*, are inevitable and restrict floral gene expression [47, 48]. In rice, a *AGL6* like gene (*OsMADS6*) determines floral meristem and floral organ identity [49]. Auxin-Responsive *OsMGH3/OsGH3–8* was modulated by *OsMADS6* and affected stamen differentiation [50]. Silencing *SlAGL6* resulted in pale green petals in plants suggesting that *SlAGL6* is involved in petal development [51]. In the present study, we found that two *AGL6* genes (NNU_08847and NNU_26581) that were mainly expressed in petal, with their expression confirmed by qRT-PCR (Fig. 6 and Additional file 1: Figure S8). Thus, these two genes were speculated to be part of the genes that regulate petal development and promote stamen conversion to petal-like organs in lotus. One type I MADS-box gene, *AGL80* (NNU_09213) expression was detected in the stamen within high-level. These findings are consistent with a prior report on this gene in *Arabidopsis* [52]. Meanwhile, it sharply declined in stamen petaloid indicating that losing NNU_09213 expression may lead to lose of stamen identity. These observations suggest that NNU_09213 is a crucial requirement for stamen determination.

As described above, we found that 15 candidate genes had medium expression in stamen petaloid compared with petal and stamen. And they are all involved in petal or stamen formation, which displayed obscure expression domains crossing the borders of petal and stamen causing the stamen petaloid phenomena.

## Conclusion

This study covers the analysis of the comprehensive transcriptomic data to provide an overview of the transcriptional changes in different floral organs in lotus cultivar ‘Fenhonglingxiao’. In addition, through pairwise comparison analyses between three floral organs, we identified 3900 DEGs likely involved in floral organ identity. The DEGs were assigned to hormone pathways and transcription factors, which were reported previously as related to flower formation. Outstandingly, the main 11 hormone related genes and 22 TFs are involved in the conversion of stamen to stamen-petaloid. We report that 29 DEGs were detected in floral organs and confirmed by qRT-PCR, and had a significant accordance with RNA-seq result. Additionally, the expression pattern of 15 candidate genes was evaluated in eight different tissues by qRT-PCR. Two of candidate genes *GH3.5* and *GATA22,* were specifically up-regulated in St vs Sp. We hypothesise that they function together in converting stamen to stamen petaloid. We identified some MADS-box genes being potential regulators of ambiguous boundaries of petal and stamen. Overall, further analysis on the mechanisms of floral formation in lotus will provide an insight and enable a deeper understanding of genetic involvement in the regulation of floral organ differentiation.

## Methods

### Plant materials

*Nelumbo necifera* cultivar ‘Fenhonglingxiao’ was used for transcriptome analysis. The cultivar has been conserved via rhizome in Wuhan Botanical Garden, Chinese Academy of Sciences (WBGCAS), Hubei Province, China for many years. The rhizomes of ‘Fenhonglingxiao’ were planted in three separate pools (3 m × 3 m) with three rhizomes per pool in the trial plot of WBGCAS on April 4th, 2016. When the flowers of ‘Fenhonglingxiao’ bloomed, petal, petaloid stamen and stamen in the same flower were sampled, respectively (Fig. 1). Meanwhile, curly leaf, unfolded leaf, flower bud, rhizome and carpel were also collected. All samples were collected at 10 A.M, snap frozen in liquid nitrogen, and stored at − 80 °C until used for RNA and DNA extraction. The petal, stamen petaloid, and stamen samples were used to construct nine libraries for RNA-seq, which were named as P1, P2, P3, Sp1, Sp2, Sp3, St1, St2 and St3, respectively.

### RNA extraction, library construction, and sequencing

Total RNAs were extracted using a RNA reagent (OminiPlant RNA Kit, CWBIO, China), and treated with RNase-free DNaseI (Thermo, Shanghai, China) to remove genomic DNA contamination. RNA integrity was evaluated, with RNA integrity number (RIN) being above 6.5 for all samples. More than 1.5 μg of total RNA per sample was used as input material for the RNA sample preparations. For each floral organ of the cultivar, these nine RNA samples were subsequently used to create cDNA libraries and Illumina sequencing by Beijing Novogene Bioinformatics Technology Co., Ltd. The cDNA libraries were sequenced using the Illumina HiSeq 2500 high throughput sequencing platform. Transcriptome assembly was accomplished using Trinity (version r20140413p1) [53] with min_kmer_cov set to 2 by default, and all other parameters set default. All clean reads data were deposited in NCBI Sequence Read Archive (SRA, https://trace.ncbi.nlm.nih.gov/Traces/sra/sra.cgi?) with accession number PRJNA417869.

### Data processing and differential expression

Sequenced reads containing adapter, poly N or low-quality sequences (Q < 20) were removed, and the remainder were termed as clean reads. All the downstream analyses were based on clean data with high quality. The clean data was uploaded in the BMKCloud (https://www.biocloud.net/) for analysis. The data was mapped to the reference genome of *Nelumbo nucifera* (China lotus 1.1) [1] using TopHat2 Software v2.0.9 [54]. The default settings were considered as tolerance parameter, enabling mismatches of less than two bases. The transcripts were annotated using Cufflinks v2.1.1 [55]. For annotations of novel genes, they were aligned using BLAST (E value < 10^− 5^) [56] to National Center for Biotechnology Information (NCBI) non-redundant protein (Nr) database, NCBI non-redundant nucleotide sequence (Nt) database, and Swiss-Prot database and the sequences with the highest similitudes were retrieved. The differentially expressed genes (DEGs) between the two samples were carried out by using DESeq package (http://bioconductor.org/packages/release/bioc/html/DESeq.html). The transcript abundance (read counts) was estimated by RSEM v1.2.15 [57]. The expression level of genes was measured by FPKM method [55]. The DEGs were identified by false discovery rate (FDR) < 0.05 and a fold change ≥2. Any genes with an adjusted *P*-value < 0.05 were assigned as differentially expressed. Expression pattern analysis using Multiple Experiment Viewer software (MeV 4.9.0, https://sourceforge.net/projects/mev-tm4/files/mev-tm4/MeV%204.9.0/) was applied. Euclidean distance was used in the Distance method and K-means for hierarchical clustering was performed.

### Gene ontology and KEGG Ortholog enrichment analysis

Based on Wallenius non-central hyper-geometric distribution [58], GO enrichment analysis of the differentially expressed genes (DEGs) was implemented by the GOseq R packages 1.10.0, which can be adjusted for gene length bias in DEGs. KOBAS (KEGG Orthology-Based Annotation System) software was used to test the statistical enrichment of DEGs in KEGG pathways (FDR ≤ 0.05).

### Quantitative real-time PCR validation of RNA-seq data

A total of 29 DEGs (log2 FC > 1 or < − 1 and FDR < 0.05) involved in floral development were chosen for validation using quantitative real-time PCR (qRT-PCR) in petal, stamen petaloid and stamen. Among the 29 DEGs, 15 genes were chosen for analysis expression pattern in eight different tissues. Primers for qRT-PCR were listed in Additional file 1: Table S2. The qRT-PCR reactions were performed on the Bio-Rad using the SYBR (BioRad, http://www.bio-rad.com/). The reaction was initiated at 95 °C for 10 s, followed by 40 cycles of 95 °C for 15 s, 60 °C for 15 s and 72 °C for 30 s. Fluorescent products were detected in the last step of each cycle. Melting curve analysis was performed at the end of 40 cycles to ensure proper amplification of target fragments. The data presented for qRT-PCR experiments are the average relative quantities from three biological replicates, where every biological replicate is the mean of three technical repeats. Relative gene expressions were normalized by comparison with the expression of lotus β-actin (NNU-24864), and analyzed using the 2^-ΔΔCT^ method. The data was indicated as mean ± SD.

## Additional files

Additional file 1: Additional figures and tables. Tables and figures in this file are referred to as **Table S1-S2** and **Figure S1-S9** in the main text. (PDF 1672 kb)

Additional file 2: 3900 DEGs. (XLSX 749 kb)

Additional file 3: Hormone related genes. (XLSX 65 kb)

Additional file 4: Floral organ homeotic genes. (XLSX 12 kb)

Additional file 5: 225 TFs. (XLSX 77 kb)

## Abbreviations

AG: AGAMOUS
AP: APETALA
DEG: Differential expression gene
FDR: False discovery rate
FPKM: Fragments per kilo base of transcript per million base pairs sequenced
MADS: MCM1, AG, DEFA and SRF
P: Petal
PI: PISITTALA
qRT-PCR: Quantitative real-time PCR
Sp: Stamen petaloid
St: Stamen
TF: Transcription factor
