# Supplementary material for: Transcriptomic analysis identifies the key genes involved in stamen petaloid in lotus (Nelumbo nucifera)
Source: BMC Genomics. 2018 Jul 27;19:554. doi: 10.1186/s12864-018-4950-0 (PMC6062958; doi:10.1186/s12864-018-4950-0)
Supplement: Supplementary file 1 — Additional figures and tables. Tables and figures in this file are referred to as Table S1-S2 and Figure S1-S9 in the main text. (PDF 1672 kb) [file 12864_2018_4950_MOESM1_ESM.pdf]

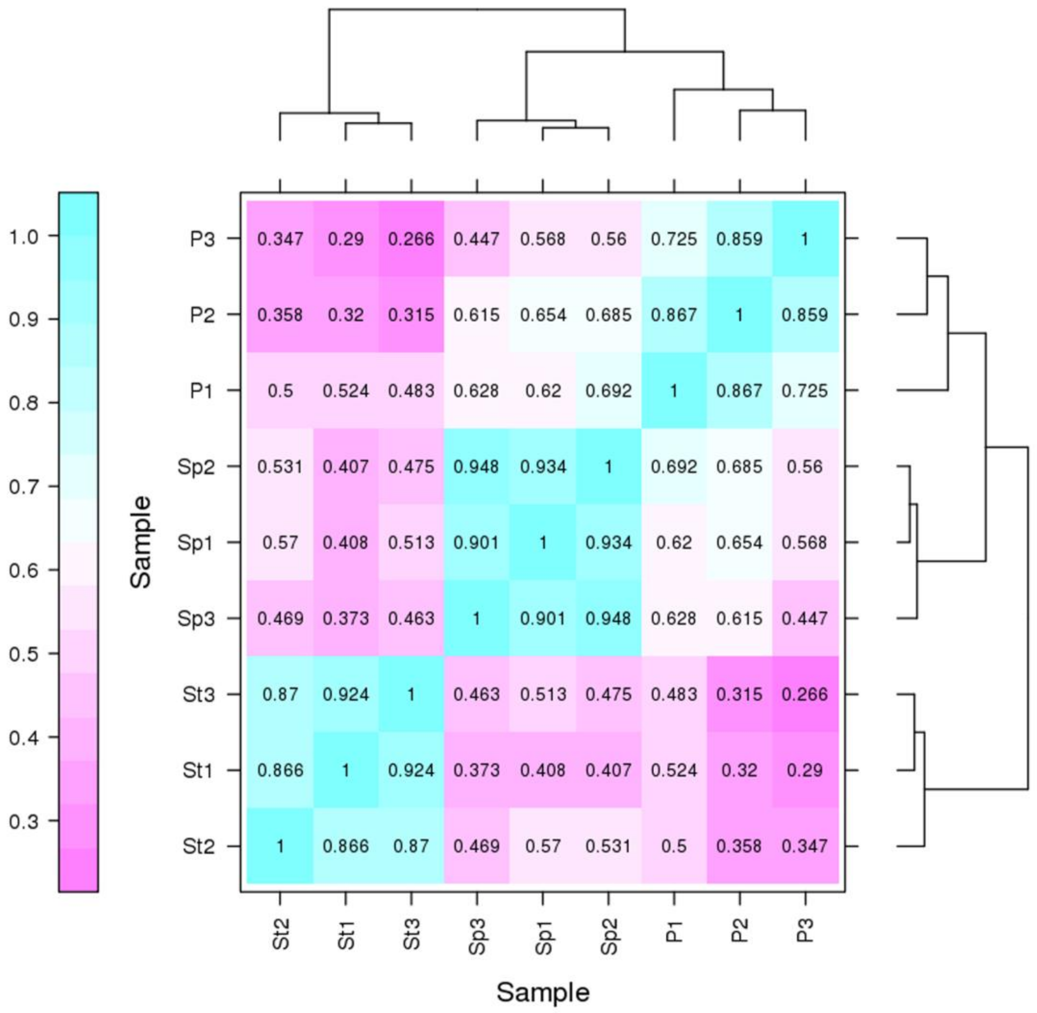

Fig. S1 Pearson's correlation coefficients for three biological replicates in different floral organ in *Nelumbo nucifera*.

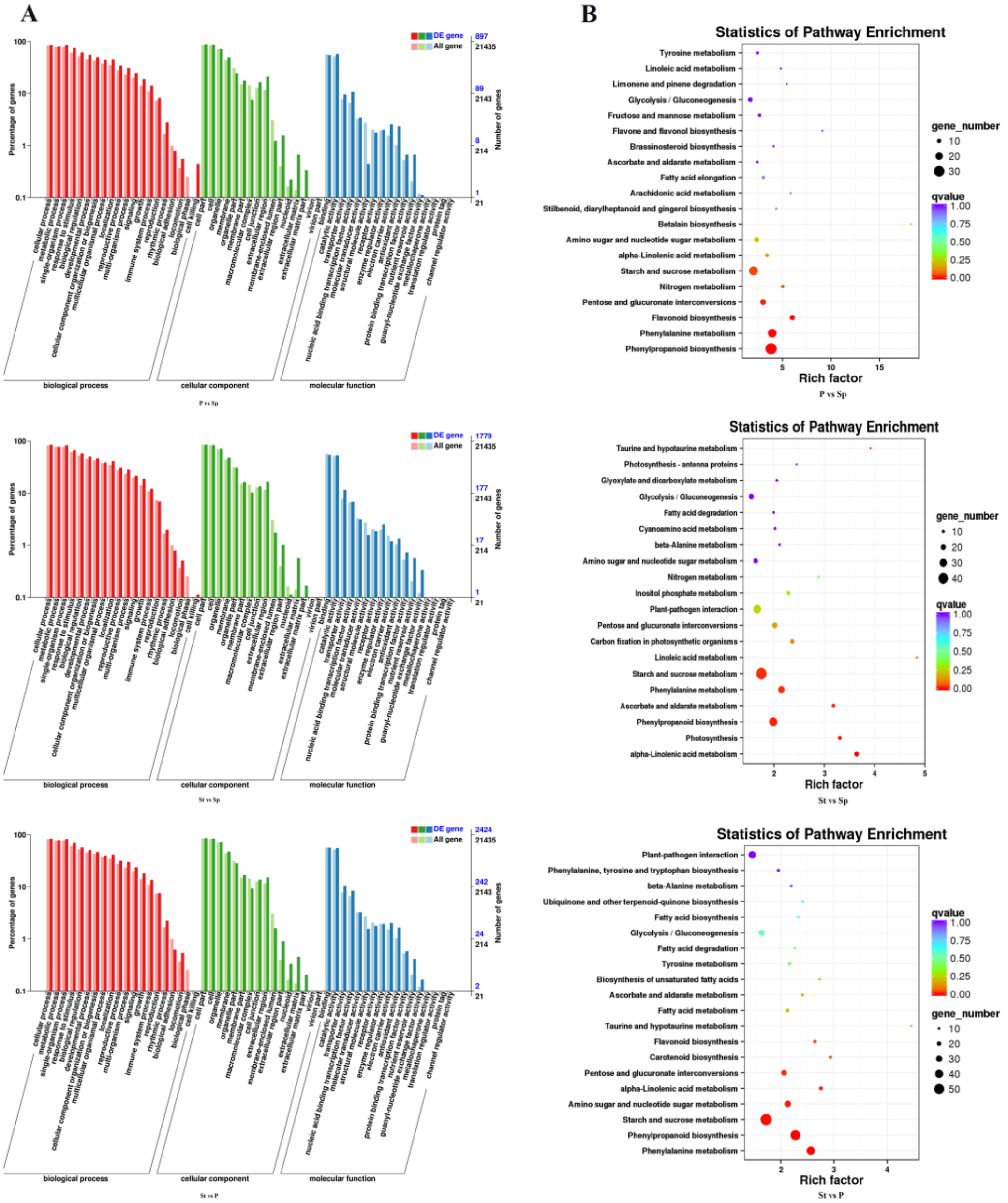

Fig. S2 GO terms (A) and KEGG pathways (B) significantly enriched in DEGs in comparisons of P, Sp and St.

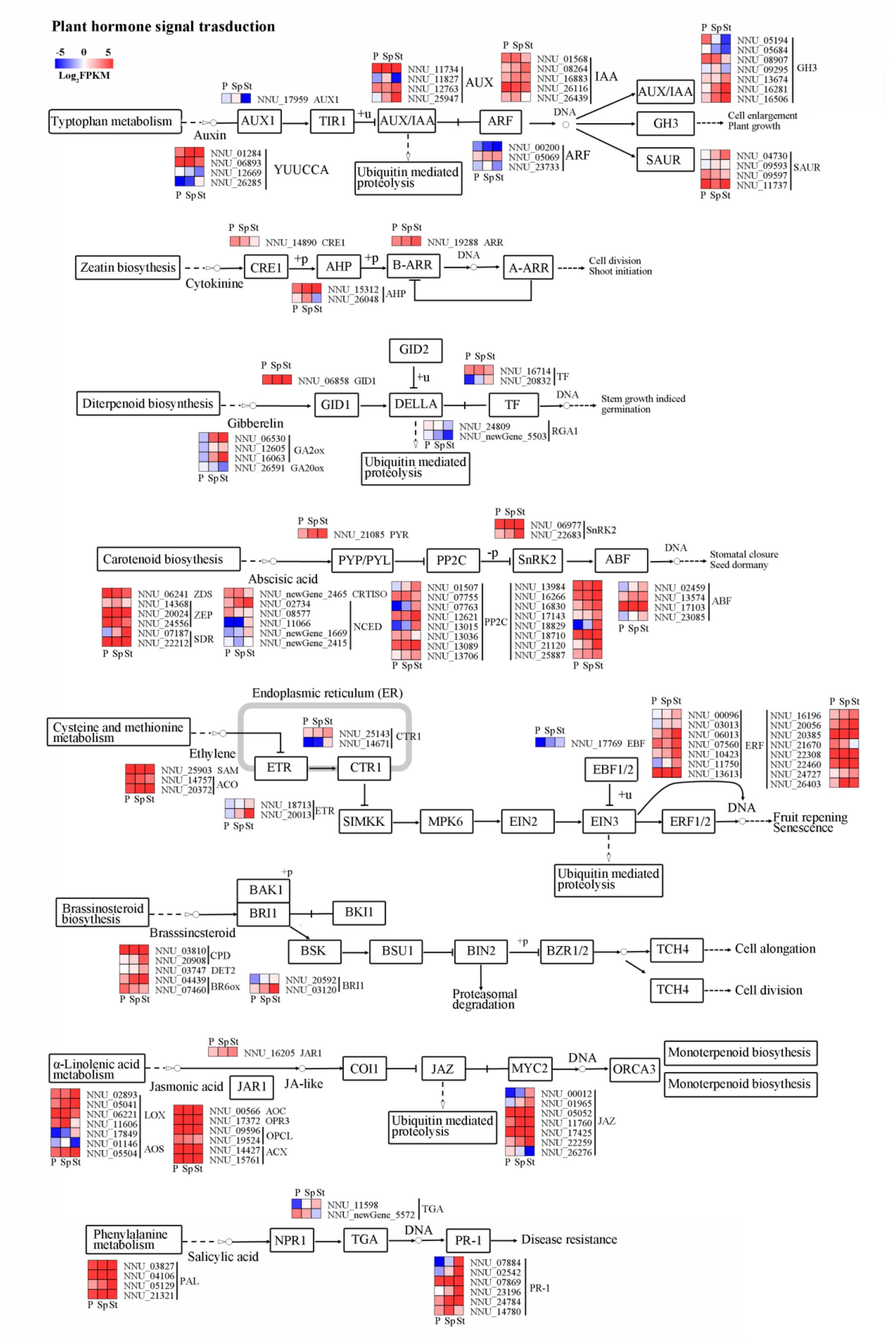

Fig. S3 Heat maps of phytohormone related genes in the three floral organs of *Nelumbo nucifera.*

The figure was revised from ko04075.

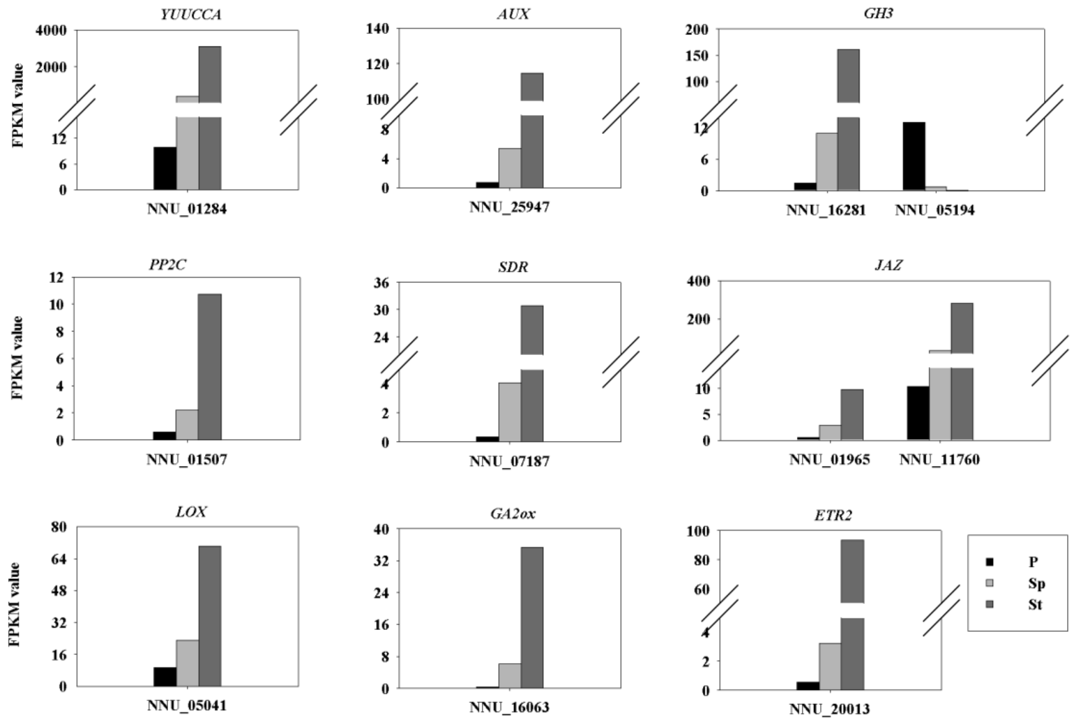

Fig. S4 Eleven phytohormone related genes.

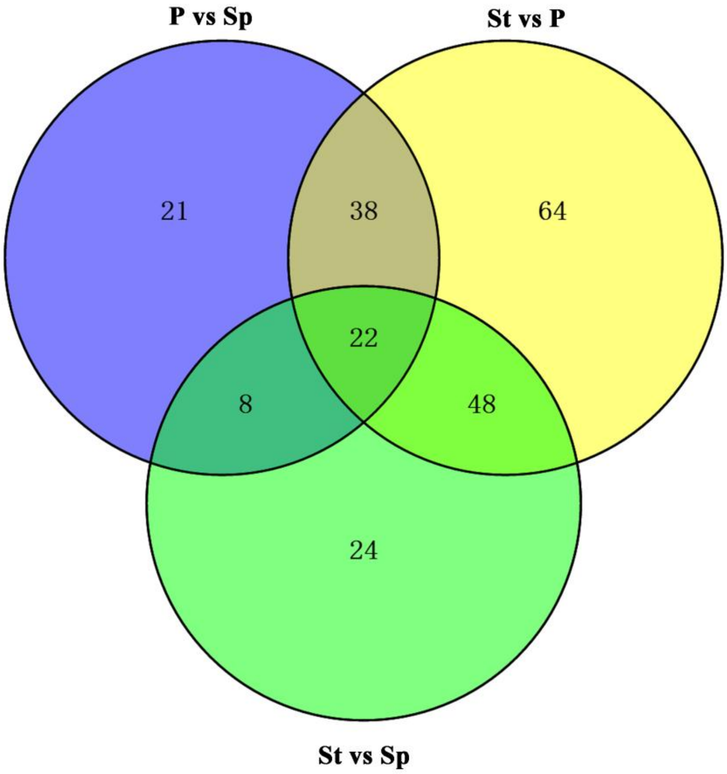

Fig. S5 The venn diagram of TFs in three pair comparison.

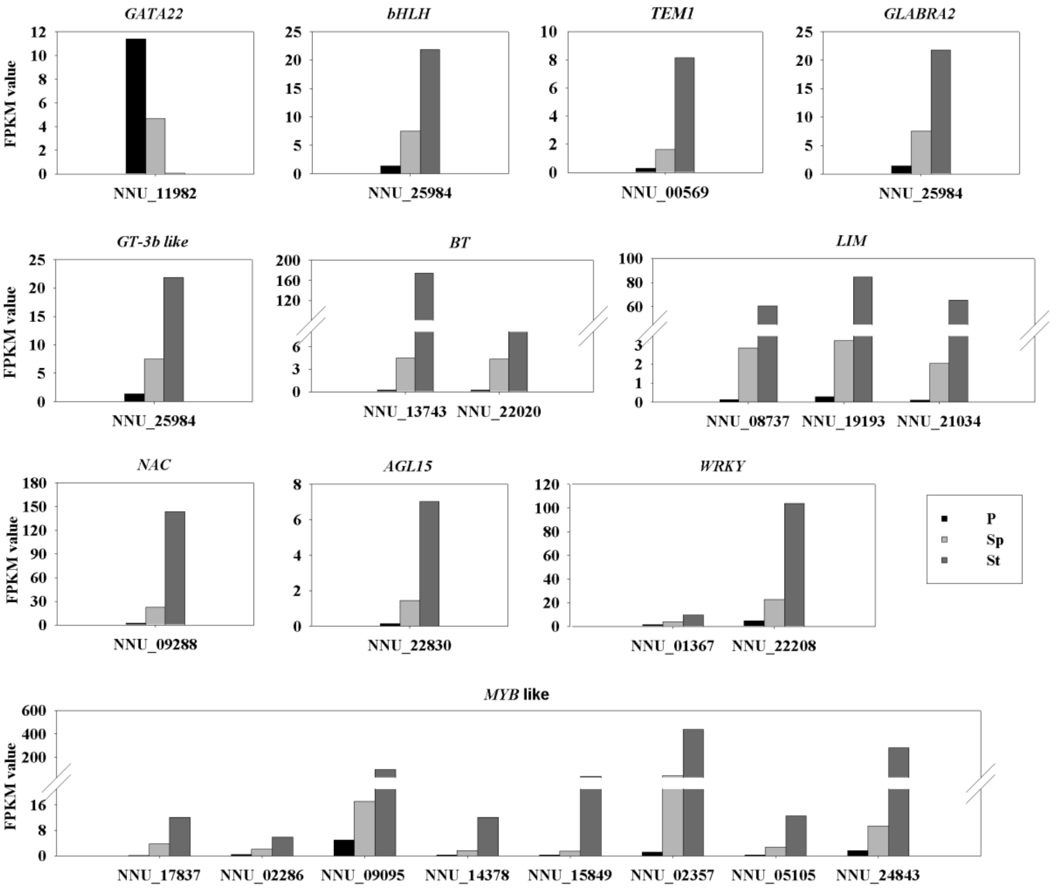

Fig. S6 22 transcription factors.

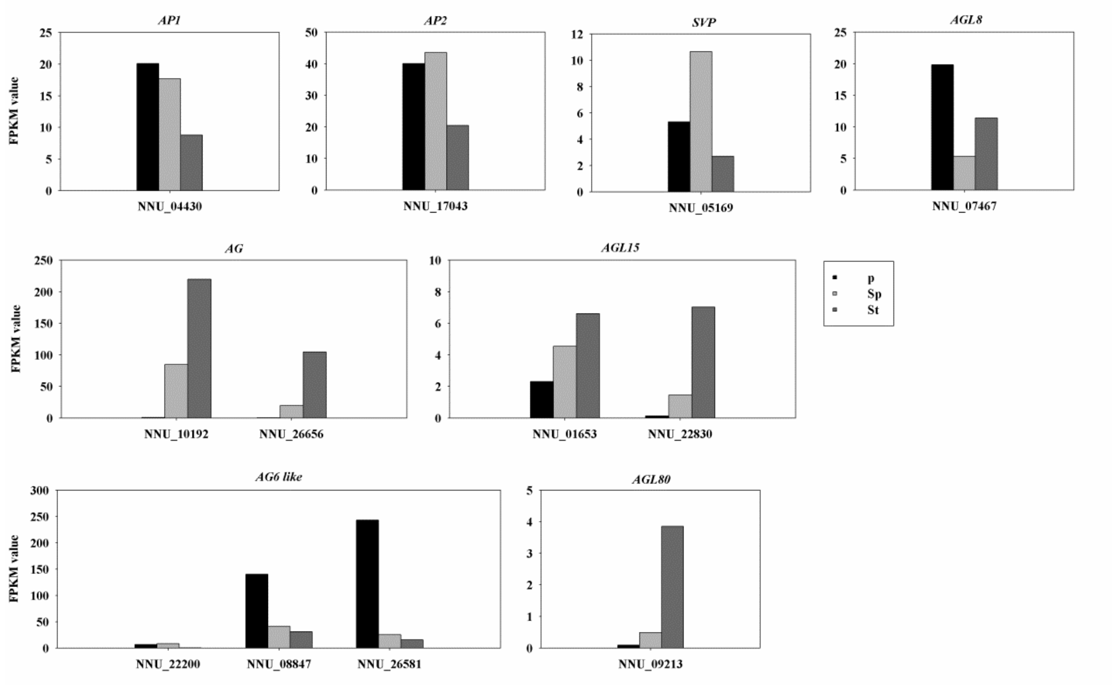

Fig. S7 Twelve floral homeotic genes.

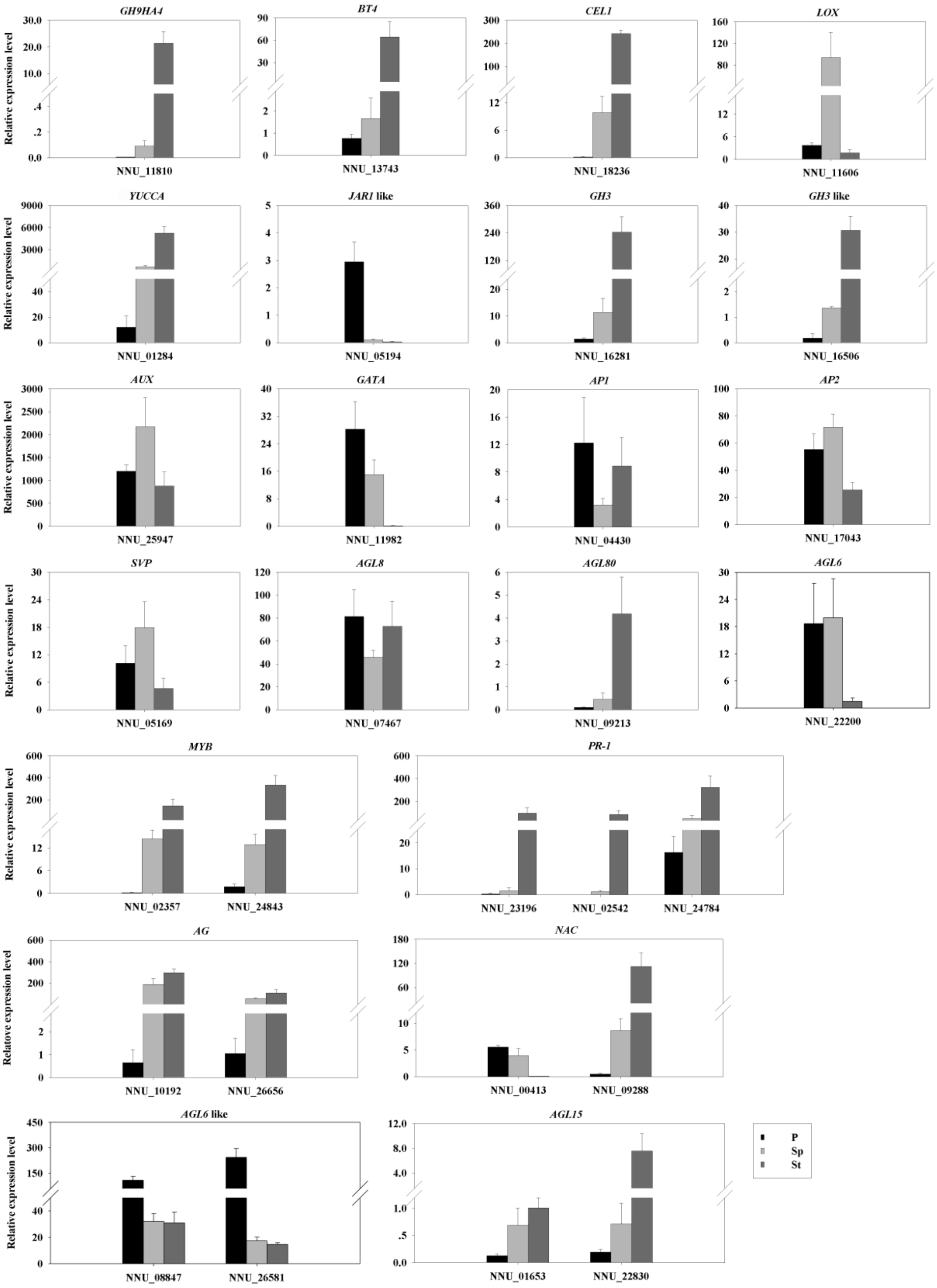

Fig. S8 Quantitative real-time PCR confirmation of 29 candidate genes at petal, stamen petaloid stamen in lotus. Relative gene expressions were normalized by comparison with the expression of lotus β-actin (NNU_24864), and using the $2^{-\Delta\Delta CT}$ method. Each gene used three biological replicates,

with three technical replicates per experiment in all qRT-PCRs. The Error bars represented the SD for three biological replicates.

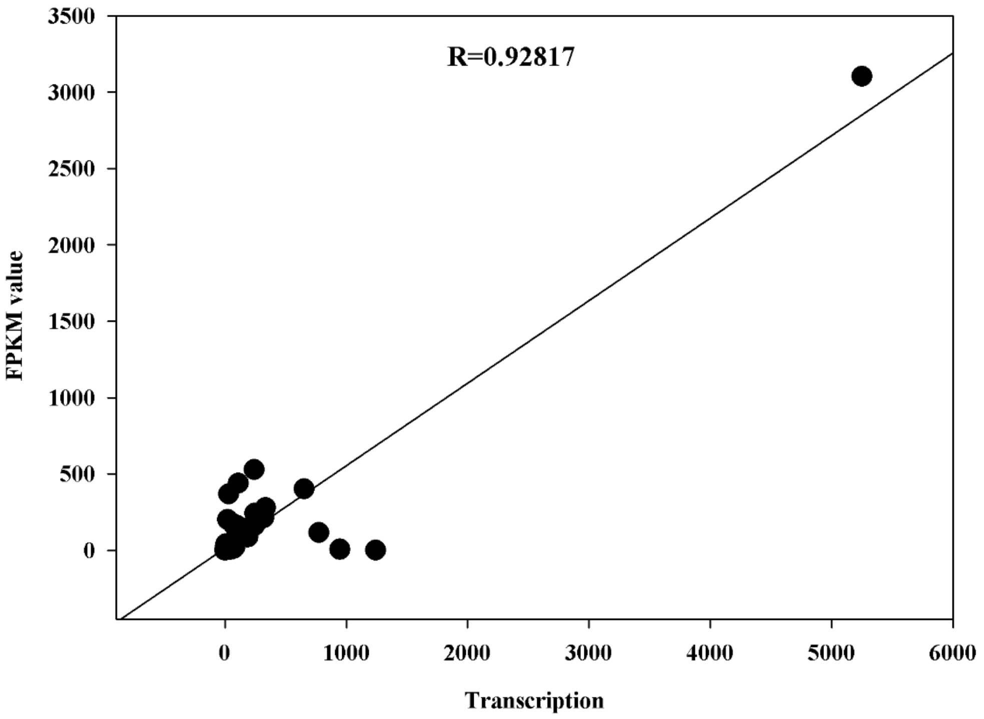

Fig. S9 The correlation between qRT-PCR result and RNA-Seq data.

Table S1. The novel transcripts annotation

Annotated Database	Annotated Number	300<=length<1000	length>=1000
**COG Annotation**	321	29	291
**GO Annotation**	758	122	629
**KEGG Annotation**	493	76	413
**Swissprot Annotation**	923	119	798
**Nr Annotation**	1835	300	1527
**All Annotated**	1836	300	1528

Table S2 Primers used to assay gene expression by qRT-PCR

Gene name	Gene ID	Forward primer (5'→3')	Reverse primer (5'→3')
β-actin	**NNU_24864**	GATGCCCTGATGAAGATCC	CCACTCAGCACAATGTTTCC
**AUX**	**NNU_25947**	AGGAGTGTGTTCAACCCCG	CACCGCTGTAGAGCCAGTCAT
**BT4**	**NNU_13743**	GTTGAGAGTTCCAGGGGGTTG	TCATCGGAGTTGGCACAGAGG
**CEL1**	**NNU_18236**	ACTCGCAGCCTCCTCCTT	CTCCCCATAACAGTTCGTCAT
**GATA**	**NNU_11982**	TCAGCGAGGTGGATGTCTTC	CAGTGATGTTGCTATTGTTGGA
**GH3.1**	**NNU_16281**	GCCTGTGATGAACCTTTAC	GCGTCTTTGTCTCGGATT
**GH3**	**NNU_16506**	CGTTGTTTTGAGCATAGATTCG	CCAGATGATTGGTGGCGTTT
**GH9A4**	**NNU_11810**	GTAAACTCCTCAACAAGGCAAAAG	CAGAATAAGAGCAATAGAATGGGC
**JAR1**	**NNU_05194**	GGTGCCGCTCTCTCAAGTTAC	ACCACATCCCCTAACCTGTATCT
**LOX**	**NNU_11606**	ACCACATCCCCTAACCTGTATCT	TGACTACTATCATCTGGGTTGCC
**MYB**	**NNU_02357**	CGAAAACACATCAAGGAAGCAG	GTCGTTAGATTCAGTGGGCAAA
**MYB**	**NNU_24843**	GACGAACACGGTAACAAGCG	GTGATGGTTTCCTCTGTCTTTG
**NAC**	**NNU_09288**	TGGGTCTTATGCCGAATCTAC	GTCAGTTTCAAGCAACGAGCC
**PRB1**	**NNU_02542**	TGGGATGATGACTGCGTTAGC	TTCTTGTTCTTCCTGGTTCGG
**PRP1**	**NNU_24784**	AAGGAGATGGTGGCGTCG	CTGGTAGGATGGAGGGCAAT
**STH21**	**NNU_23196**	GCTGAAATCTGCTTGCTACGAG	ACATTCCAGTCATCCCATCCTT
**YUCCA10**	**NNU_01284**	ATCTCCCCTACTTCGCCGTG	CGATGATTGGATACTTGCCGT
AG	**NNU_10192**	TGGAGGGTAGATTGGAGAAAGG	TGGCGAGAGTAATGGTGGTTG
AG	**NNU_26656**	GCAGAAAAGGGAGATTGAGTTA	GTGGTTGGGTTCCAGTAGATT
AGL6	**NNU_22200**	TACCAGTTTGCCAATCCCG	ATGCCTGTGCCTTGCCTC
AGL6 like	**NNU_26581**	ATGGGAAGAGGACGAGTAGAG	GTCGCAGAGCACAGAAAGC
AGL6 like	**NNU_08847**	TTTCTTTCTCGTGGTCGGCTAT	AACCGTTCCTCCGCTTGG
AGL8	**NNU_07467**	AAGCGGAGGAACGGATTGC	CCCTTGGTGGAGAAGACGAT
AGL15	**NNU_01653**	CGGCGGATGATGGGTAAAG	CAATCGGAGATAAGGTGGCA
AGL15	**NNU_22830**	AGAGGGAAGATTGAAATAAAGAGG	CACTGCTGGAAAAGATAATGAGAG
AGL80	**NNU_09213**	AATAGCAGTGCCGTGTTCC	ATTGATTTCCGCTTGGGTTA
AP1	**NNU_04430**	TAGCACAAGAACGAGGATGG	CAGTTTGGACAATGAAGGGA
AP2	**NNU_17043**	CATACGACAGAGCAGCCATAA	GAGCCTCCCATCTTCCACAT
SVP	**NNU_05169**	GCGGTTGAGCAAGGAAGTT	CGCCTGACAAGCACGAAGT
